# Does FDG PETCT have a predictive value for neoadjuvant chemotherapy response in nonmetastatic breast cancer?

**DOI:** 10.1007/s11845-024-03856-6

**Published:** 2024-12-18

**Authors:** Ender Dogan, Safak Yildirim Disli, Esra Asik, Seyhan Karacavus, Feyyaz Ozdemir

**Affiliations:** 1 Department of Medical Oncology, Kayseri City Education and Training Hospital, Kayseri, Turkey; 2 Department of Medical Oncology, Karadeniz Technical University, Trabzon, Turkey; 3 Department of Nuclear Medicine, Kayseri City Education and Training Hospital, Kayseri, Turkey

**Keywords:** Breast cancer, Complete response, Neoadjuvant treatment

## Abstract

**Background:**

A pathological complete response (pCR) rate after neoadjuvant chemotherapy (NAC) is important for the prognosis of early-stage breast cancer. The prediction of an NAC response plays a key role in managing neoadjuvant treatment.

**Aims:**

The aim of this study is to investigate the predictive value of the baseline PETCT FDG (F-18 fluoro-deoxy-glucose (FDG) positron emission tomography/computed tomography) SUVmax (the maximum standardized uptake value) for pCR after NAC in early-stage breast cancer.

**Methods:**

The patients who performed PETCT before NAC were included in this retrospective study. The basal PETCT SUVmax values were divided into two categories based on the cutoff points of ≥ 8.77 or < 8.77, namely the low SUV max group and the high SUV max group. These two groups were compared according to the general characteristics. The impact of the PETCT SUVmax values on pCR was determined with logistic regression analyses.

**Results:**

One hundred forty-eight patients who performed PETCT before NAC were included in this retrospective study. Eighty-one patients were in the low SUV max group and 67 patients were in the high SUVmax group. The pCR trended toward a higher rate in the high SUVmax group than in low SUVmax group but it was not statistically significant (*p* = 0.052). The baseline PETCT SUVmax value was an independent predictive factor for pCR. (*p* = 0.025).

**Conclusion:**

PETCT SUVmax may be a factor for the predicting complete response to neoadjuvant treatment in early-stage breast cancer.

## Background

Neoadjuvant chemotherapy is the preferred initial therapy in most local advanced and some early breast cancers [1]. The pathological complete response after neoadjuvant chemotherapy is associated with better disease-free and overall survival rates in breast cancer patients [2]. The prediction of the NAC response is important to manage neoadjuvant treatment. It helps us to make decisions on the best upfront therapy. It also helps with avoiding ineffective treatments and chemotherapy toxicities [3].

Neoadjuvant chemotherapy is more effective in more aggressive breast cancer tumors. Receptor status is one of the indicators of the aggressiveness of breast cancer. Triple-negative and HER2-positive tumors are more aggressive than luminal tumors and they have a favorable response to neoadjuvant chemotherapy [4]. Other indicators of aggressiveness are high tumor grades and high proliferation rates [4]. A high SUVmax in a PETCT scan is associated with the high proliferation and the aggressiveness of breast cancer [5].

PETCT is a widely used method in breast cancer. A PETCT scan shows the glycolytic metabolism of tumor cells in breast cancer [6]. A high FDG SUVmax is associated with high glucose uptake [7]. Past reports have revealed that FDG PETCT correlated with some prognostic indicators such as histological grade, histological type, tumor size, estrogen receptor status, and progesterone receptor status [8]. High PETCT SUVmax is associated with biological aggressiveness [9]. Asaoka et al. reported that the pCR is associated with the aggressive biology of breast cancer [10]. These studies suggested that the patients who had high baseline FDG SUVmax in their PETCT scans had a high probability of a pCR. Pathological indicators may not be enough to predict the pCR because it is evaluated only using biopsy specimens not whole tumor tissue in pathological examinations. PETCT scans could evaluate intratumoral heterogenity better than a needle biopsy. A PETCT examination may show a high FDG uptake in parts of the high-grade tumor tissue unless the biopsy specimen shows low-grade disease. The aim of this study is to investigate the predictive value of baseline PETCT FDG SUVmax of complete pathological responses after neoadjuvant treatment of early-stage breast cancer.

## Patients and method

The patients diagnosed with early-stage breast cancer who were admitted to Kayseri City Education and Training Hospital and Karadeniz Technical University Hospital were retrospectively reviewed. One hundred forty-eight patients were included in this study. All patients had a basal PETCT scan before undergoing neoadjuvant chemotherapy treatment and they all received neoadjuvant chemotherapy treatment.

We retrospectively recorded the age, gender, histological type, menopausal status, tumor size, grade, lymph node status, receptor status, NAC response status (complete and noncomplete response), and type of surgery (mastectomy or breast conserving therapy) from the hospital archives. We retrospectively reviewed the PETCT SUVmax before and after neoadjuvant chemotherapy treatment. The difference of the SUVmax values in post- and pre-treatment (Difprimer PETCT SUVmax) was calculated with ΔSUV max = 100 x (post-treatment SUVmax – baseline SUVmax)/baseline SUV max formula.

The basal PETCT SUVmax was divided into two categories based on the cutoff points of ≥ 8.77 or < 8.77, namely the low SUVmax group and the high SUVmax group, using ROC curve analysis [area under the curve, 0.602 (0.496–0.687); specificity, 0.611; sensitivity, 0.552; *p* = 0.049].

These two groups were compared according to general characteristics. No invasive tumor on the tumor bed was defined as a complete pathological response.

Molecular classification was defined as luminal A, luminal B, triple negative, and HER2 enriched. The luminal A group had high ER (estrogen receptor) and/or PR (progesterone receptor) expressions, HER2 negative, and low ki67 (< 20%). The luminal B group had low ER and/or PR positivity, or PR negativity, HER2 negative, and high ki67 (> 20%). The HER2 enriched group had HER2 + 3 IHC positive or HER2 SISH positive. The triple negative group was the hormone and HER2 negative group [11].

This study was approved by the Kayseri City Education and Research Hospital (19/14.03.2024).

## Statistics

For statistical analyses of the study data, “IBM SPSS Statistics for Windows. Version 25.0 (Statistical Package for the Social Sciences, IBM Corp., Armonk, NY, ABD)” was used. Descriptive statistics were frekans and % for categorical variables, mean ± sd, and median (IQR) for continuous variables. Independent sample *t* test or the Mann–Whitney *U* test was used for independent variables according to normality test. Ki-kare $\left({\chi }^{2}\right)$ test was used to compare categorical variables. ROC (Receiver Operating characteristics Curve) curve analysis was used to determine cutoff value for PET CT primary SUVmax. Logistic regression analyses were used to find out the association between complete response (pCR ve pnCR) and other explanatory variables. Statistical significance was regarded as *p* < 0.05.

## Results

We included 148 patients in the study. The median age of the patients was 49 years old. Other general characteristics are shown in Table 1.

**Table 1:** General characteristics

Characteristics	General population		Low SUV max (*n* = 81)	High SUV max (*n* = 67)	*P*
	Mean $\pm$ sd	Median (IQR)	Mean $\pm$ sd	Mean $\pm$ sd	
**Age**	49.77 ± 11.46	49.00 (18.75)	51.32 ± 11.91	47.89 ± 10.68	0.093**#**
**Ki67 preop**	35.93 ± 22.24	30.00 (30.00)	29.86 ± 19.06	43.39 ± 23.70	** < 0.001#**
**Tm diameter**	31.48 ± 14.25	28.50 (18.00)	28.60 ± 15.87	34.88 ± 11.27	**0.008‡**
**NLR (neutrophil/lymphocyte ratio)**	2.26 ± 1.16	1.97 (1.20)	2.14 ± 1.16	2.40 ± 1.15	0.070**#**
Difprimersuv	− 81.25 ± 24.96	− 89.77 (30.00)	− 79.49 $\pm$ 25.09	− 83.59 ± 24.88	0.515**#**
	**f (n)**	**%**	**f (%)**	**F (%)**	**P**
**Age group**					
Below age 50 years old	83	56.1	41 (51)	42 (63)	0.141*
Over age 50 years old	65	43.9	40 (49)	25 (37)	
**Menopausal Status**					
Premenopause	78	52.7	39 (48)	39 (58)	0.222*
Postmenopause	70	47.3	42 (52)	28 (42)	
**Molecular subgrup**					
Luminal A	20	13.5	16 (20)	4 (6)	
Luminal B	33	22.3	17 (21)	16 (24)	0.090*
HER2 enrich	56	37.8	30 (37)	26 (39)	
Triple negative	39	26.4	18 (22)	21 (31)	
**Grade**					
1	16	10.8	12 (17)	4 (7)	
2	64	41.8	37 (53)	25 (43)	0.038*
3	50	33.7	21 (30)	29 (50)	
Unknown	20	13.8			
**Estrogen receptor**					
Positive	91	61.5	56 (69)	35 (52)	0.035*
Negative	57	38.5	25 (31)	32 (48)	
**Progesteron receptor**					
Positive	81	54.7	50(62)	31 (46)	0.074*
Negative	66	44.5	31 (38)	35 (54)	
Unknown	1	0.8			
**Estrogen receptor10**					
< 10	62	41.9	28(35)	34 (51)	0.047*
> = 10	86	58.9	53 (65)	33 (49)	
**Stage**					
1	2	1.4	2 (3)	0	
2	97	65.5	53 (65)	44 (66)	0.425*
3	49	33.1	26 (32)	23 (34)	
**Axillary metastasis**					
Yok	31	20.9	19 (24)	12 (18)	0.426*
Var	117	79.1	62 (76)	55 (82)	
**Surgery**					
Breast conserving therapy	59	39.1	36 (45)	23 (34)	0.240*
Mastectomy	89	61.9	45 (55)	44 (66)	
**Pathological response**					
Complete response (pCR)	58	39.1	26 (32)	32 (48)	0.052*
Noncomplete response (pnCR)	90	61.9	55 (78)	35 (52)	

^‡^Independent sample *t* test

^#^Mann–Whitney *U* test

^*^ $${\chi }^{2}$$ test

*p* < 0.05 statistically significant

The Ki67 preop values and tumor diameters were statistically significantly lower in the low SUVmax group than in the high SUVmax group (*p* < 0.001, *p* = 0.008, respectively). Grade 1 and 2 tumors were statistically much more in the low SUV max group and grade 3 tumors were statistically more prevalent in the high SUVmax group (*p* = 0.038). The estrogen receptor positivity levels were statistically much more in the low SUVmax group than in the high SUV max group (*p* = 0.035). The estrogen receptor higher positive 10% group was statistically more prevalent in the low SUVmax group than in the high SUVmax group (*p* = 0.047). Complete response was higher in the high SUV max group than in the low SUVmax group but this result was not statistically significant (*p* = 0.052). Other variables were similar between the two groups.

We demonstrated that the tumor diameter, HER2, ER, and PECT SUVmax variables are independent predictors for complete response in the logistic regression analyses. Low tumor diameter, HER2 positivity, ER negativity, and high PETCTSUVmax values predict the increasing possibility of pCR. Age is not an independent predictor factor for pCR (Table 2).

**Table 2:** Logistic regression analyses

	B	S.E	*p*-value	Exp(B)	95% CI for EXP(B)
Age	− 0.012	0.019	0.529	0.988	0.952–1.026
Tm diameter	− 0.062	0.018	**0.001**	0.940	0.907–0.974
HER2(negative/positive)	2.225	0.471	** < 0.001**	9.252	3.667–23.278
ER (negative/positive)	− 1.693	0.462	** < 0.001**	0.184	0.074–0.455
PETCT SUVmax(low/high)	1.033	0.459	**0.025**	2.811	1.142–6.917

## Discussion

The prediction of response to NAC plays a key role in deciding which patients are more amenable to NAC. Some pathological indicators show us that the tumor biology and can possibly predict the NAC response. Imaging methods such as FDG PETCT scans help us to provide information about tumor behaviors, and therefore about the NAC response as well. Theoretically, PETCT FDG SUVmax is associated with high proliferation rates in tumor cells. Therefore, it is associated with the NAC response, particularly the pCR. In our study, we revealed that high SUVmax is associated with high pCR rates, but this value was not statistically significant. Also, we found that the high baseline SUVmax was statistically independent predictive indicator of the pCR in early-stage breast cancer patients who received NAC treatment.

In literature, there are conflicting results about the prediction of the complete response to NAC according to the baseline PETCT SUV max values. Sengoz et al. reported that baseline PETCT FDG SUVmax is not associated with the NAC response [6]. They showed that ΔSUVmax (difference of SUVmax values in post- and pre-treatment) and post-treatment SUVmax were significantly different between responder and nonresponder groups. They found that the ΔSUVmax value is the only predictive marker for the neoadjuvant response. In this study, they defined responders as Miller Payner grade 4 and 5 groups. We defined the pCR only as Miller Payner 5. In our study, the complete response was higher in the high baseline SUVmax group than in the low SUVmax group. This result was not statistically significant, but it was nearly significant. In our study, the number of patients were more than in this study. In their study, there were only 30 patients. As the number of patients increases, it nearly approaches statistical significance. In a study from Champion et al., they found that the baseline PETCT SUVmax tended to be higher in the complete response group [12]. However, this association was not significant, which is similar to our study. They included only 23 patients in their study. Sobhi et al. reported on a study that analyzed PETCT and MRI effectiveness as pCR predictors [13]. They included 40 patients in their study and they analyzed the differences in the baseline SUVmax after two cycles of chemotherapy and the SUVmax change between the two cycles between the complete and noncomplete response groups. In their study, in the complete response group, the baseline PETCT SUVmax was significantly higher than in the noncomplete group. However, the change in SUVmax after NAC treatment was similar in the complete and noncomplete response groups. They analyzed the response to NAC earlier on than our study. We analyzed the NAC response after the completion of neoadjuvant treatment. Gallivanone et al. evaluated the effect of PETCT parameters on the pCR [14]. They found that none of the PET features were significantly correlated with the pCR to NAC treatment. They concluded that PET does not play a role in predicting the NAC response. The number of patients in their study was low with 38 patients. In our study, high SUVmax predicted the complete response and tended to be higher in the complete response group than in the noncomplete response unless it was not statistically significant. In a large trial with 124 patients, it was demonstrated that radiomics analysis based on pretreatment FDG PET/CT scans predicted NAC response [15]. This result is compatible with the results of our study.

In the high SUVmax group, ki67 was significantly higher than in the low SUVmax group in our study. Ki67 is a well-known proliferation marker in breast cancer [16]. The association between ki67 and PETCT SUVmax has been controversial. Many studies have suggested that the PETCT SUVmax correlated with high pretreatment ki67 levels. A study reported that evaluating PETCT parameters and ki67 values from Surov et al. They concluded that there were moderate correlations between FDG PET SUV max and Ki-67 values in breast cancer (0.40) [17]. Lee et al. demonstrated a moderate correlation between the SUVmax and ki67 values as well [18]. They suggested that ki67 is correlated with FDG PETCT, but a moderate correlation did not allow for using it in clinical practice.

In our study, tumor size, grade, and estrogen negativity were statistically higher in high the SUVmax group than in the low SUVmax group. Abdeal et al. demonstrated that tumor size is correlated with high PETCT SUVmax. Similar to our study, they also found that SUVmax of primary tumors was higher in patients who had negative estrogen receptor status and high-grade tumors [19]. Kaide et al. reported that tumor size and nuclear grade were significantly positively correlated with baseline PETCT SUVmax. They also reported that estrogen negativity was associated with a high FDG uptake [20]. In another study, they found that FDG uptake was higher in T2 tumors than in T1 tumors, in ER-negative tumors than in ER-positive tumors, in PR-negative tumors than in PR-positive tumors, in ki67 higher tumors than lower tumors, and in high-grade tumors than in low grades tumors [21]. These findings are consistent with our results. Although the SUVmax was higher in PR-negative tumors than PR-positive tumors in our study, it was not statistically significant. Goorts et al. reported that the most important predictor for the pCR in early-stage breast cancer was tumor diameter. They concluded that a smaller tumor diameter had significantly higher pCR rates than a high tumor diameter. They also found that high-grade, positive HER2 positivity, and negative ER and PR receptor statuses were significant predictors of higher pCR rates [22]. In another study, they found that tumor size, grade, Ki67 expression levels, histological subtypes, chemotherapy protocol, and baseline PETCT parameters, including SUV_max_, SUV_peak_, SUV_mean_, MTV (metabolic tumor volüme), and TLG (total lesion glycolysis) were significantly associated with the pCR [23].

Baseline PETCT SUV max cutoff values are different in literature. Lee et al. found the cutoff value for the baseline PETCT primer SUV max as 9.55 in ROC curve analyses [24]. In their study, the area under the curve was 0.703, sensitivity was 87.5%, and specificity was 69.3% for this cutoff. In another study, the cutoff value of the baseline PETCT SUV max was 3.66 [23].

Our study is one of the largest studies that evaluated the prediction of complete response according to baseline PETCT FDG SUVmax. However, our study had several limitations. First, this study had a retrospective design. Second, there was heterogenity of molecular subtypes as the response differed according to the receptor groups. Thus, we were unable to evaluate responses to different chemotherapy regimens based on the histological subtype. Another limitation is the lack of sufficiency of PETCT for intratumoral heterogenity. Intratumoral heterogenity could be the reason of noncomplete response despite high baseline PETCT SUVmax.

In conclusion, baseline PETCT SUV max may predict the complete response to NAC treatment in early-stage breast cancer. Even though it was not statistically significant, the high PETCT SUVmax tended to be higher in patients who completely responded to neoadjuvant treatment. These results should be carefully interpreted with other pathological indicators. Also, large prospective trials are needed to adapt these results to clinical practice.

## Data Availability

Requests for materials should be addressed to E.D.
